# Using Molecular Markers to Characterize Productivity in Chinese Hamster Ovary Cell Lines

**DOI:** 10.1371/journal.pone.0075935

**Published:** 2013-10-17

**Authors:** Raihana Z. Edros, Susan McDonnell, Mohamed Al-Rubeai

**Affiliations:** School of Chemical and Bioprocess Engineering and Conway Institute of Biomolecular and Biomedical Research, University College Dublin, Dublin, Ireland; Institute of Molecular and Cell Biology, Biopolis, United States of America

## Abstract

Selection of high producing cell lines to produce maximum product concentration is a challenging and time consuming task for the biopharmaceutical industry. The identification of early markers to predict high productivity will significantly reduce the time required for new cell line development. This study identifies candidate determinants of high productivity by profiling the molecular and morphological characteristics of a panel of six Chinese Hamster Ovary (CHO) stable cell lines with varying recombinant monoclonal antibody productivity levels ranging between 2 and 50 pg/cell/day. We examined the correlation between molecular parameters and specific productivity (*q_p_*) throughout the growth phase of batch cultures. Results were statistically analyzed using Pearson correlation coefficient. Our study revealed that, overall, heavy chain (HC) mRNA had the strongest association with *q_p_* followed by light chain (LC) mRNA, HC intracellular polypeptides, and intracellular antibodies. A significant correlation was also obtained between *q_p_* and the following molecular markers: growth rate, biomass, endoplasmic reticulum, and LC polypeptides. However, in these cases, the correlation was not observed at all-time points throughout the growth phase. The repeated sampling throughout culture duration had enabled more accurate predictions of productivity in comparison to performing a single-point measurement. Since the correlation varied from day to day during batch cultivation, single-point measurement was of limited use in making a reliable prediction.

## Introduction

The slow progression of recombinant protein production in the biopharmaceutical industry is mainly due to the low productivity of host cell lines [1]–[3]. One major challenge in generating high-producing cell lines is the lengthy selection process which typically takes six to nine months by the traditional method of cloning by limiting dilution [4].

However, over several rounds of amplifications, these high producers are prone to copy number loss, which introduces variability in productivity levels [5], [6]. The resulting productivity instability has been characterized by the combination of an imbalance in chromosome number, an absence of TTAGGG*_n_* sequence, the rearrangement of targeted genes to transcriptionally inactive sites and the methylation of promoters at CpG dinucleotides [7]–[9]. Regardless, it seems evident from earlier studies that high copy numbers will not necessarily result in high productivity [9]–[11], which suggests that the cause of decreased productivity is not restricted to the genetic level.

While several research groups [12]–[20] found that high productivity was strongly associated with an abundance of recombinant transcript level, Smales et. al., (2004) did not observe this correlation. The lack of correlation was postulated to be due to the limited resources of processing and secretory apparatus during the folding and assembly step that primarily takes place in the endoplasmic reticulum (ER) [21]. Certain transcription regulators, such as X-box binding protein (X-BP1) and activating transcription factor 4 (ATF4) and ER proteins, including binding protein (BiP), protein disulphide isomerise (PDI) and glucose-regulated proteins 94 (GRP94), have been shown to influence the ER expansion during protein synthesis and thus affecting the secretion rate of antibody [22]–[33]. In the case of LC and HC mRNA abundance, the assembly and folding of antibody can be limited by the low expression of the ER proteins. It has been shown that low expression of ER proteins in a small ER volume can result in antibody aggregation [34].

The decreased productivity could also result from a slow secretion rate [17]. The saturation of the secretory pathway has been regarded as one possible bottleneck limiting the efficient protein trafficking involved in exocytosis mediated by soluble N-ethylmaleimide-sensitive factor attached protein receptor (SNAREs) (reviewed by [35]. Overexpression of SNAREs, SNAP-23, VAMP8 and Munc18b in various types of mammalian cells has resulted in increased secretion capacity which consequently led to an improvement in the cell productivity [36], [37].

The identification of limiting factors during the choreography of protein synthesis and secretion described above has indirectly identified several features that could potentially help to predict productivity. A few studies have successfully developed prediction methods of production stability based on several molecular markers e.g., human cytomegalovirus major immediate early promoter/enhancer (hCMV-MIE) methylation and transgene copy numbers [38] and intracellular antibody and apoptotic markers, such as caspase 3 and annexin V [39]. Even so, there still remains a need for more markers that could possibly serve as tools to predict productivity level and to select high productivity cell lines.

This study was initiated to provide productivity markers with high sensitivity and high specificity for CHO cells producing monoclonal antibody with the aim of improving detection of high producers. Although several studies have been reported, few are of sufficiently high level of evidence to permit solid conclusions. We designed experiments to obtain results with high reproducibility and interpretability. In these experiments we identified markers based on their correlation with productivity, as statistically evaluated by Pearson correlation coefficient analysis. In order to verify the reliability of the identified molecular markers we monitored their levels throughout growth phase. This approach has highlighted the importance of making daily measurements; repeated experiments showed that this ensures more reliable and consistent analysis to identify the causes of clonal variation in mammalian cell cultures than the single-point measurement.

## Materials and Methods

### Cell lines and maintenance

This study employed six GS-CHO cell lines (referred to as CL38, CL47, CL76, CL150, CL160 and CL164) producing cB72.3 IgG4 monoclonal antibody with varying levels of productivity. Cell lines were kindly provided by Lonza Biologics (Slough, UK). The suspension-variant derivative of CHO-K1 (CHOK1SV) cell lines were generated by transfection with glutamine synthetase (GS) expression vector, pcB72.3 containing LC and HC, each driven by the hCMV-MIE promoter [40], [41]. Cells were maintained in CD-CHO medium supplemented with 25 µM methionine sulphoxamine (MSX). Experiments were conducted in 125 ml shaker flasks (SCHOTT North America, Inc., Elmsford, NY, USA), incubated at 37°C and agitated at 140 rpm. All experiments were conducted using three biological replicates.

The six cell lines were numbered according to the specific productivity in descending order with [1] referring to most productive cell line and [6] referring to the least productive cell line. These cell lines are referred to as CL 47[1], CL 76[2], CL 150[3], CL 164[4], CL 38[5] and CL 160[6] throughout the work. Also, based on the specific productivity, CL 47[1] and CL 76[2] were categorised as high producers, while CL 150[3], CL 164 [4] and CL 38[5] as medium producers; and CL 160[6] as low producer.

### Cell biomass determination

Cell dry weight was determined as previously described [16]. Briefly, petri dishes (Nalge Nunc International, Rochester, NY, USA) were dried in an oven at 70°C for at 48 hours before being cooled down to room temperature. Each dish was weighed in triplicate. During the growth phase, 5.0×10^6^ cells were removed from each flask and centrifuged. The pellet was then washed with sterile-filtered PBS and centrifuged again. The cell pellet was dispersed with deionized water before being transferred to a pre-weighed petri dish. The culture dishes were kept in an oven for 48 hours at 70°C before being weighted.

### Determination of total intracellular protein content

The total protein content was determined using the QuantiPro™ BCA Assay Kit (Sigma-Aldrich, St Louis, USA). 3.0×10^6^ were removed and centrifuged before being washed once with sterile-filtered PBS. Next, a mixture of CelLytic™M Cell Lysis Reagent (Sigma-Aldrich) and protease inhibitor cocktail (Sigma-Aldrich) were added to disrupt the cells. The samples were mixed by vortexing for about one minute, centrifuged at 15 000 g for 15 minutes at 4°C, and stored at −20°C until analysis. The supernatant was kept at −20°C until analysis.

### Flow cytometric analysis of cell number, viability and cell size

Cells suspension (490 µl) was taken in a FC tube, to which 10 µl propidium iodide (PI) solution was added from the stock solution (0.5 µg/ml). Mixed by gentle shaking and analyzed immediately for cell numbers and viability using Cell Lab Quanta™ SC flow cytometry (Beckman Coulter Inc, CA, USA) equipped with an argon laser (488 nm). Red fluorescence (PI) was collected using a 670 nm long pass filter. Analysis was undertaken by loading an appropriate protocol for the acquired parameters: Electronic volume (EV), log side scatter (SS) and PI integral; 10000 observations were collected for analysis. The evaluation of cell number was achieved by gating areas in the EV vs log SS dot plot in which living cells and dead cells appear. The cell volume was concurrently measured based on the changes in electrical resistance produced by nonconductive particles suspended in the saline solution. We used the following equation to calculate the specific growth rate, *μ*:
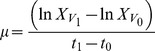
where *X_V1_* and *X_V0_* symbolize the viable cell density at time points *t_1_* and *t_0_* respectively.

### Determination of antibody concentration

Enzyme-linked immunosorbent assay (ELISA) was performed to determine the monoclonal antibody (MAb) concentration secreted by the six cell lines, as described previously [42]. The primary antibody was the monoclonal anti-human IgG Fc (Sigma-Aldrich) and anti-human kappa light chain horseradish peroxidase (HRP) conjugates (Sigma-Aldrich) were used as the secondary antibodies. The concentration of antibody in the samples was determined using o-phenylenediamine (OPD; Sigma-Aldrich) as a substrate. The following formula was employed to calculate *q_p_* as previously described [16], based on cell number (µg/10^6^ cell/day), total protein (µg/µg/day), cell dry weight (µg/µg/day), and cell volume (µg/µm^3^/day):
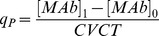
where [MAb] represents the antibody concentration at a particular time while cumulative viable cell time (CVCT) is the sum of the individual areas given by
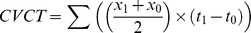
where, *x_0_* represents cell number, biomass, protein content or cell volume at a particular time *t_0_* and *x_1_* represents them at the elapsed time of *t_1_*.

### Analysis of intracellular HC and LC polypeptides by flow cytometry

2.0×10^6^ cells were removed, centrifuged and the resulting cell pellet was washed with cold PBS. The cell pellet was resuspended in 4% paraformaldehyde and was incubated in the dark for 20 minutes at 4°C. Then, the cells were resuspended in 70% methanol and incubated again at 4°C for 1 hour. The cells were centrifuged and washed with 1% BSA in cold PBS. The cells were divided equally; for the HC polypeptide, the cells were stained with anti-human (FC specific) FITC conjugate, while LC polypeptide was detected using anti-human kappa LC FITC conjugate. The samples were incubated at 4°C for 30 minutes. After incubation, the cells were washed with cold PBS and resuspended in cold PBS. The HC and LC fluorescence emissions were measured with a 530/30 bandpass (BP) filter [16], [43], [44].

### Flow cytometric analysis of ER and Golgi content

The 5 mg of BODIPY® FL C_5_-Ceramide (Molecular Probes by Invitrogen, Carlsbad, CA, USA) was dissolved in deionised water. This stock solution contained equal moles of sphingolipid and BSA and was diluted a 100-fold to 5 µM prior to use. The ER and Golgi apparatus content in the six cell lines was analyzed by using Cell Lab Quanta™ SC flow cytometry (Beckman Coulter Inc.) as previously described [26]. Cells were removed and stained with BODIPY® FL C_5_-Ceramide dye to a final concentration of 5 µM. The sample was incubated at 4°C for 15 to 30 minutes. The sample was then washed with sterile-filtered cold PBS or cold medium before being incubated at 37°C for 20 minutes. The ER and Golgi apparatus fluorescence signals were collected at 515 nm and 620 nm respectively after excitation by perpendicular laser at 488 nm.

### RNA and DNA isolation

The cell pellet recovered from 3.0×10^6^ cells that have been harvested at growth phase was kept at −80°C until analysis. DNA isolation was carried out by using QIAamp DNA Blood Mini Kit (QIAGEN, Valencia, CA, USA), following a method for mammalian cells described in the manufacturer's instructions. For RNA isolation, 3.0×10^6^ cells were removed, centrifuged and washed twice in PBS. The sample was resuspended in RNA*later* and stored at −80°C until analysis, at which point it was centrifuged at 500 g for 10 minutes to remove the RNA*later*. RNA isolation was carried out using RNeasy Mini Kit (QIAGEN) according to the manufacturer's instructions. The amount of DNA and RNA was determined using a NanoDrop ND-1000 UV-Vis Spectrophotometer (Nanodrop Technologies, Wilmington, DE, USA). An Agilent Bioanalyzer (Santa Clara, CA, USA) was used to check the RNA integrity.

### Absolute quantification of HC and LC gene copy numbers

Plasmids were kindly supplied by Lonza Biologics (Slough, UK). HC and LC genes were incorporated separately to different plasmids with sizes of 4270 bp and 3592 bp respectively. DNAse free water was added to vials containing 5 µg of dried plasmids to a final concentration of 0.05 µg/µl plasmid stock solutions. These stock solutions served as standards, each containing HC and LC plasmids with 1.1×10^12^ and 1.4×10^12^ copy number respectively. Each stock solution was serially diluted to generate standards from 2.0×10^1^ to 5.0×10^4^ copy number for HC and 1.9×10^5^ to 6.0×10^6^ copy number for LC. Following the serial dilution, the plasmids were amplified by qPCR under the following thermal cycling conditions: 2 minutes at 50°C, 10 minutes at 95°C to activate the enzyme, followed by 40 cycles of 15 seconds at 95°C for denaturation and 1 minute at 58°C for annealing and extension using ABI PRISM 7900HT Sequence Detection System (Applied Biosystem, Foster City, CA, USA). Standard curves were generated by plotting *C_T_* values against log of plasmid copy number for both HC and LC (*R^2^* = 0.99; data not shown).

The qPCR master mix was prepared by mixing SYBR® Green PCR Master Mix (Applied Biosystem), DNase-free water, sense and anti-sense primers (HC, sense primer: 5′ AGCCCAAGGATACCCTGATGA 3′, anti-sense primer: 5′ TGCTGATGGGTTTTCTCGATGC 3′; LC, sense: 5′ CCCAGCAGATTCAGCGGCAGCG 3′ and anti-sense primer: 5′ GTCCTGCTCGGTCACGCTCTCCTGG 3′). The master mix was prepared without DNA template (standards and samples) and was aliquoted with volume sufficient for three technical replicates. 2 ng of DNA template was added to the tube and the mixture was mixed by gentle aspiration. The qPCR was performed using an ABI PRISM 7900HT Sequence Detection System (Applied Biosystem) using the following thermal cycling conditions as described above. The logarithm of the HC and LC copy numbers in 2 ng of DNA in each cell line were calculated from the *C_T_* values given by the following equation:
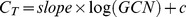
where *c* is the intercept of standard curve.

### Relative quantification of HC and LC mRNA expression level

cDNA was prepared from 2 µg total RNA that had been treated with DNAse (Sigma-Aldrich) following the instruction from the kit. DNAse I Reaction Buffer and 1 µl DNAse I (1 U/µl) was added to the sample prior to 10 minutes of incubation at room temperature. To stop the reaction, 25 mM EDTA solution was added and the samples were heated at 70°C for 10 minutes. The sample was placed on ice until cDNA synthesis. The cDNA was synthesized using SuperScript II RT Kit (Invitrogen Corporation (CA, USA) following the manufacturer's instructions. Random primers, dNTP mix (Invitrogen) and RNase-free water were added to the sample. The sample was heated to 65°C for 5 minutes and was chilled on ice. 5× First Strand Buffer and 0.1 M DTT (Dithiothreitol, impairs disulphide bond formation) was added to the sample before being incubated at 25°C for 2 minutes. The reaction was started by adding SuperScript II RT into the sample prior to sequential incubation at 42°C and 70°C for 50 and 15 minutes respectively. The *C_T_* values that were obtained from the qPCR analysis were normalized to that of a housekeeping gene, 18S ribosomal RNA (18SrRNA) and CL 160[6] as a calibrator using *2^−ΔΔCT^* method as previously described [45].

### mRNA degradation

The experiment was conducted following a procedure described previously [16]. Exponentially growing cells were removed and reseeded in fresh CD-CHO growth medium at a seeding density of 2.0×10^5^ cells/ml. After 30 hours of incubation, 1 mg/ml actinomycin D (Sigma-Aldrich) was added to each culture replicate to a 5 µg/ml final concentration. Cells were harvested after 3 hours and 10 hours of Actinomycin D treatment time. These samples were washed once with cold PBS and the pellet was resuspended in RNA*later* and stored at −80°C until analysis. The expression level of HC and LC was quantified according to the protocol described above. The rate of mRNA decay, *k_d_*, was obtained from the slope of semi-log graph of HC and LC concentrations against time. The mRNA half-life, *t_1/2_*,was calculated as follows:
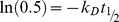



### Statistical analysis

#### Pearson correlation coefficient

Pearson correlation coefficient, *ρ*, was calculated to measure the strength of association between two variables, *X* and *Y*. The correlation coefficient between X and Y can be calculated using the following equation [46]:
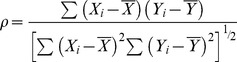
where 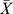 and 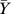 represent the mean of each variable while 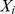 and 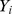 represent the raw data of *X* and *Y*. The *ρ* values vary between +1 to −1. In this case, the correlation analysis was performed by using SigmaPlot 11.0 (Systat Software, Inc., San Jose, CA) to determine the strength of the relationship between specific productivity and molecular markers at 95 percent confidence intervals.

#### Student t-test and Analysis of Variance (ANOVA)

Experimental data are presented as means ± standard deviation, with n representing the number of biological replicates. Differences between levels of molecular markers at day three of batch culture were tested using Student's t-test while ANOVA was used to test the differences of molecular markers during growth phase (day one to day five) at 95 percent confidence intervals. The statistical analysis was performed by using SigmaPlot 11.0 (Systat Software, Inc., San Jose, CA).

## Results

### Growth, viability and productivity of the six GS-CHO cell lines

**Figure 1:**
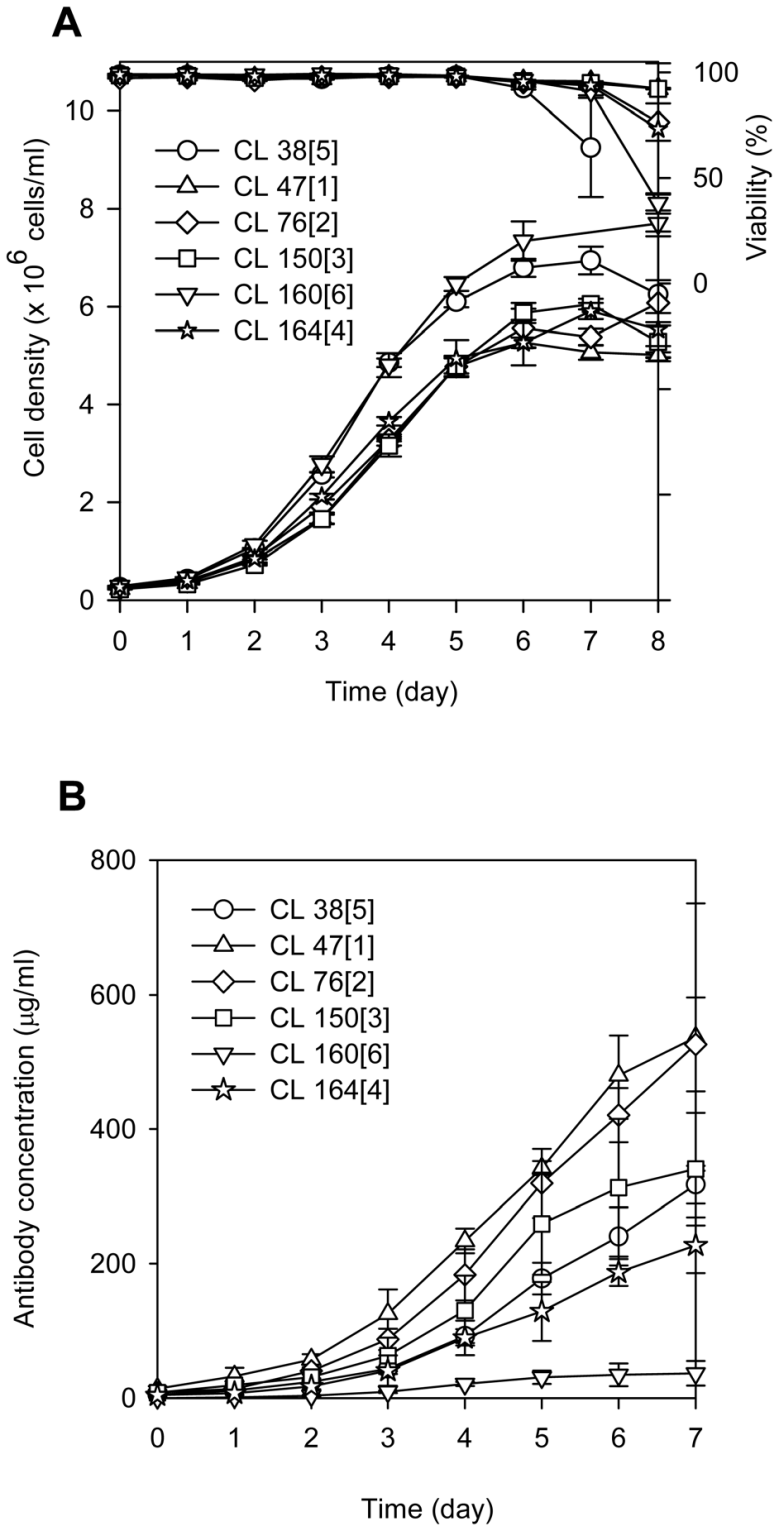
Cell growth, viability and MAb concentration. Growth and viability curves (A) of GS-CHO cell lines analyzed by flow cytometry and (B) MAb concentration batch culture.

Figure 1 shows the growth, viability and antibody concentration profiles of the six cell lines in the batch cultures. The corresponding growth characteristics of these cell lines are tabulated in Table 1. The growth and antibody production in the six cell lines were significantly different (P-values<0.05) as computed using Student t-test analysis.

**Table 1:** Growth characteristics and cell productivity.

Cell line	Maximum viable cell density (10^6^ cells/ml)P-value<0.001	Specific growth, μ_net_ (per day)P-value<0.001	Doubling time, τ_d_ (h)P-value<0.001	Maximum product concentration (µg/ml)P-value = 0.019	Specific productivity, *q_p_* (pg/cell/day)P-value = 0.001
47[1]^a^	5.27±0.10	0.63±0.01	26±0.55	575.24±137.55	50.54±3.18
76[2]	6.22±0.23	0.63±0.02	26±0.68	526.24±70.14	47.23±4.65
150[3]	6.05±0.11	0.65±0.01	26±0.25	362.17±61.57	38.73±7.27
164[4]	5.88±0.15	0.63±0.01	27±0.54	280.71±50.70	21.23±0.24
38[5]	7.16±0.52	0.68±0.01	24±0.16	421.13±100.83	18.34±1.64
160[6]	7.70±0.26	0.67±0.01	25±0.29	52.08±0.77	2.91±1.45

The growth characteristics and cell productivity of the respective cell lines determined from day 1 to day 5 of batch culture. The symbol ± represents the standard deviation, which was calculated between the three biological replicates.

a Cell lines were numbered according to the secretion rate as shown in parenthesis. P-values were calculated based on Student t-test analysis at 95% confidence intervals.

Following a typical growth curve, the cell density of CL 160[6] and CL 38[5] were higher than CL 47[1], CL 76[2], CL 150[3] and CL 164[4]. The six cell lines maintained their viability at more than 95% during the growth phase before gradually entering the death phase. Having the slowest growth rates, CL 47[1] exhibited the highest total antibody, followed by CL 76[2], CL 38[5], CL 150[3], CL 164[4] and CL 160[6]. The specific productivity for the six cell lines is tabulated in Table 1. CL 47[1] is again shown to be the most productive cell line followed by CL 76[2], CL 150[3], CL 164[4], CL 38[5] and CL 160[6].

High specific productivity usually results in slower growth rates, which can be seen in CL 47[1]. CL 47[1] (*q_p_* = 50.54 pg/cell/day) exhibited the lowest specific growth rate, μ of 0.63 day^−1^, compared to CL 160[6] (μ = 0.67 day^−1^; *q_p_* = 2.91 pg/cell/day). Two of the medium producers, CL 76[2] and 164[4] exhibited similar specific growth rates as CL 47[1]. Meanwhile, the growth rate of CL 38[5] was observed closer to that of CL 160[6]. These observations are consistent to the doubling time. Based on Pearson correlation coefficient analysis, a negative correlation between specific growth rates and specific productivity (ρ = −0.56; P-value = 0.014) was observed. The negative correlation could be associated with the increase in metabolic burden in the high-producing cell lines as reported previously [9], [24], [47]–[50].

### Specific productivities accounting for physiological characteristics

**Figure 2:**
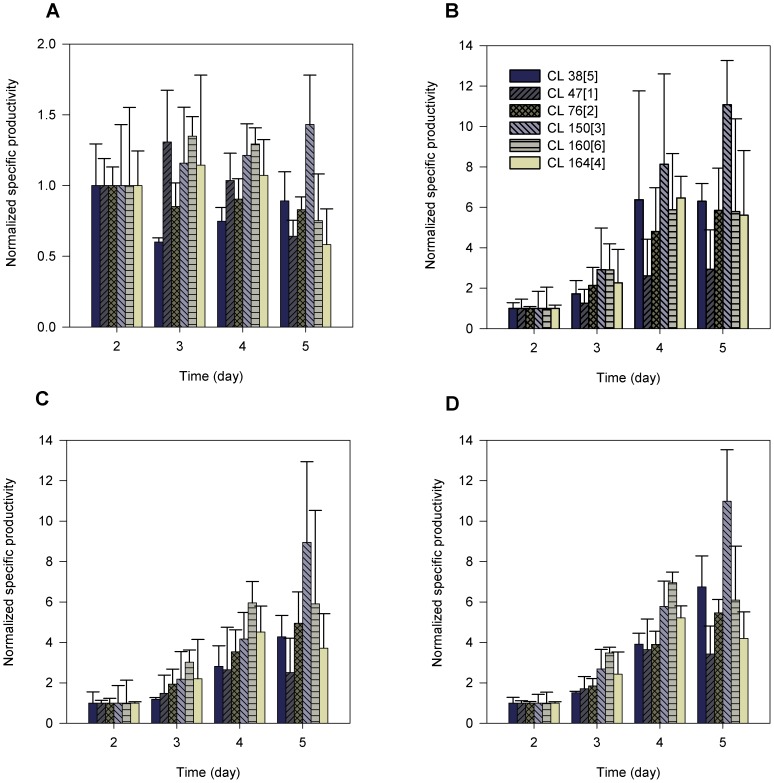
Specific productivities accounting for physiological characteristics. Specific productivities calculated based on cell number (A; P-value = 0.053), total protein content (B; P-value<0.001), biomass (C; P-value<0.001), and cell volume (D; P-value<0.001). The data were normalized based on day two culture. Error bars symbolize the standard deviation as calculated between three biological replicates. P-values were calculated based on ANOVA at 95% confidence intervals.

In order to study the change of monoclonal antibody production with regard to the change in the physiological characteristics in the six cell lines, we calculated the specific productivity based on the cell number, total protein content, biomass, and cell volume. This calculation method was employed previously [16] to elucidate the relationship between cell growth and the increase in cell productivity in a cytostatic system. The calculated specific productivities at day 2, 3, 4 and 5 were normalized to the specific productivity calculated at day 2 of culture. These data represent the amount of monoclonal antibody secreted per unit of mass (total protein content or biomass) or volume relative to day two of culture. These specific productivities are indicated in Figure 2.

Figure 2A represents the specific productivity calculated based on the cell number. There was no general trend describing the change of these specific productivities in the six cell lines from day 2 to day 5. Relative to day 2, the specific productivity of CL 76[2] and CL 38[5] considerably decreased at day 3. In CL 76[2], this level increased to a maximum value before decreasing again as the cells progressed to day 5. However, in CL 38[5], the specific productivity increased proportionally until day 5. As for CL 47[1], CL 150[3], 164[4] and CL 160[6], the specific productivity considerably increased at day 3. Unlike CL 150[3], the specific productivity in CL 47[1], CL 160[6] and CL 164[4] decreased proportionally until day 5. The specific productivity of CL 150[3] increased throughout the growth phase from day 2 to day 5 of batch culture.

Figure 2B represents the specific productivity calculated based on the total protein content. As the culture progressed from day 2 to day 5, the specific productivity based on total protein content of CL 76[2] and CL 150[3] was increased. Between these cell lines, CL 150[3] showed the highest degree of increment that was almost 11-fold (relative to day 2 culture) as the culture progressed from day 4 to day 5. On the contrary, the specific productivity of CL 47[1], 38[5] and 160[6] was increased until day 4 and remained at the same level until day 5. This was also observed in CL 164[4] but the level was decreased from day 4 to day 5. Interestingly, the changes of specific productivity that were calculated based on the total protein were similar to that of biomass (Figure 2C) and cell volume (Figure 2D). Comparing the production of monoclonal antibodies per biomass, total protein and volume, CL 150[3] outperformed CL 47[1] with 1.5- to 3.6-fold, 2.3- to 3.8-fold and 1.6- to 3.2-fold greater than CL 47[1] respectively. This could be due to the fact that CL 150[3] exhibited relatively lower total protein content (1.73-fold; data not shown), and biomass (1.33-fold; data not shown) than CL 47[1]. This finding is consistent with CL 160[6] indicating that CL 150[3] and CL 160[6] produced more monoclonal antibody per unit mass than medium producers with lower production capacities. When the cultures reached the stationary phase (days 4 and 5), the specific productivity of CL 47[1] became saturated (days 3 to 4 = 2.6-fold; days 4 to 5 = 2.9-fold). This did not occur in CL 150[3] whereby the specific productivity increased to 11-fold as the culture progressed from day 4 to day 5.

### Variations at the molecular level causing differences in *q_p_*

**Figure 3:**
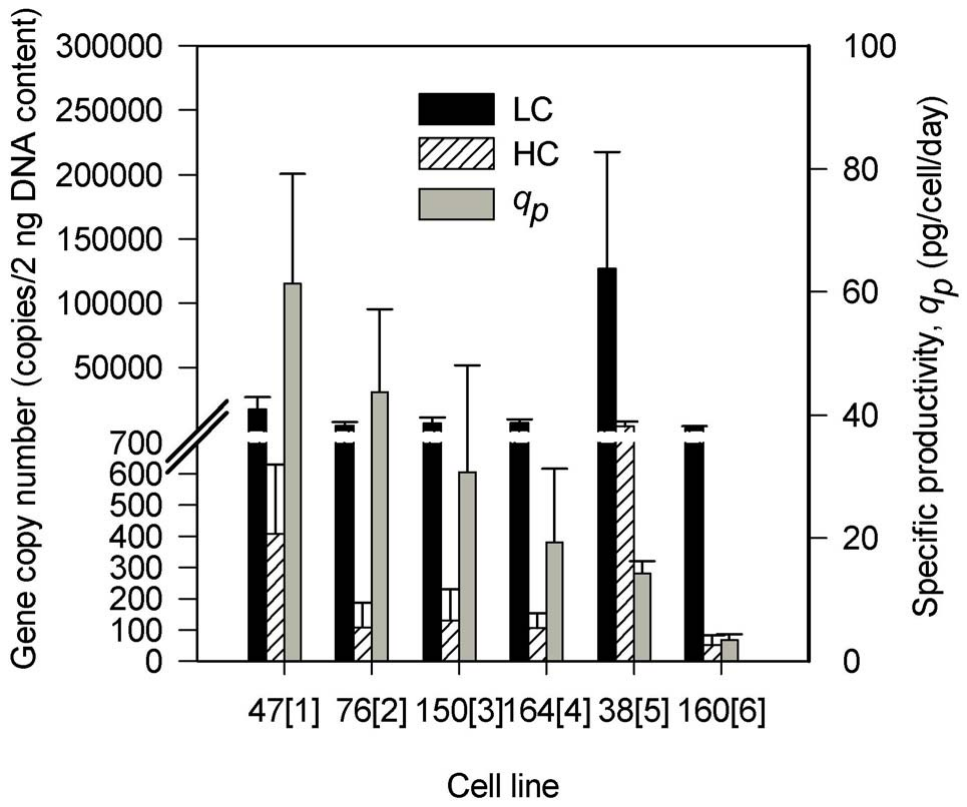
Gene copy numbers of heavy chain (HC) and light chain (LC). qPCR analysis of HC and LC gene copy numbers (GCNs) using SYBR Green chemistry at growth phase (day 3). Error bars symbolize the standard deviation calculated from three biological replicates. Student t-test analysis showed that the difference between the levels of HCs GCNs in the six cell lines was found to be insignificant (P-value = 0.05).

In attempting to understand whether the increase in the levels of HC and/or LC GCNs could result in enhanced specific productivity, we estimated the GCNs of HCs and LCs at the growth phase. Figure 3 illustrates the GCNs of HCs and LCs for the six GS-CHO cell lines. In general, LCs displayed higher GCNs in all cell lines than HCs. We also observed that both LC and HC GCNs in CL 38[5] were the highest among the six GS-CHO cell lines. However, the specific productivity of CL 38[5] did not reflect this. CL 47[1], exhibiting the highest specific productivity, indicates almost the same LC GCN but had a higher HC GCN than CL 160[6]. These GS-CHO cell lines varied in the HC and LC GCNs which resulted in a lack of correlation between specific productivity and GCN with Pearson correlation coefficients of both HC and LC, ρ = −0.26 and −0.24 (P-value>0.05) respectively. Therefore, we concluded that high GCN is not always accompanied by high specific productivity.

The LC to HC GCN ratios in the six cell lines are demonstrated in Table 2. LC GCNs are present abundantly in all six cell lines, though to a lesser extent in CL 47[1] and CL 38[5] than in the others. The LC to HC GCN ratios between CL 47[1] and CL 160[6] were not reflected in the specific productivity. Besides, one of the high producers, CL 76[2] and certain medium producers such as CL 150[3] and CL 164[4] showed higher ratios than CL 47[1].

**Table 2:** mRNA and polypetides parameters for 6 CHO cell lines.

Cell line	LC∶HC GCN ratioP-value = 0.002	LC∶HC mRNA ratioP-value = 0.002	LC∶HC polypeptide ratioP-value<0.001	HC Half-life (hr)P-value = 0.002	LC Half-life (hr)P-value<0.001
47[1]	44.80±3.23	0.87±0.07	2.12±0.01	1.85±0.14	4.28±0.10
76[2]	54.42±3.19	1.18±0.13	1.49±0.01	1.38±0.21	5.33±0.13
150[3]	59.86±12.81	1.14±0.19	1.02±0.07	1.66±0.34	4.15±0.22
164[4]	70.77±5.75	1.22±0.37	2.78±0.01	1.41±0.16	4.28±0.16
38[5]	29.20±4.15	0.87±0.10	1.05±0.04	1.09±0.40	3.40±0.17
160[6]	71.92±14.81	1.00±0.00	1.25±0.11	2.31±0.11	8.35±0.04

HC to LC chain ratios for GCN, mRNA, and intracellular polypeptides, and mRNA half-lifes of GS-CHO cell lines determined at growth phase. P-values were calculated based on Student t-test analysis at 95% confidence intervals.

**Figure 4:**
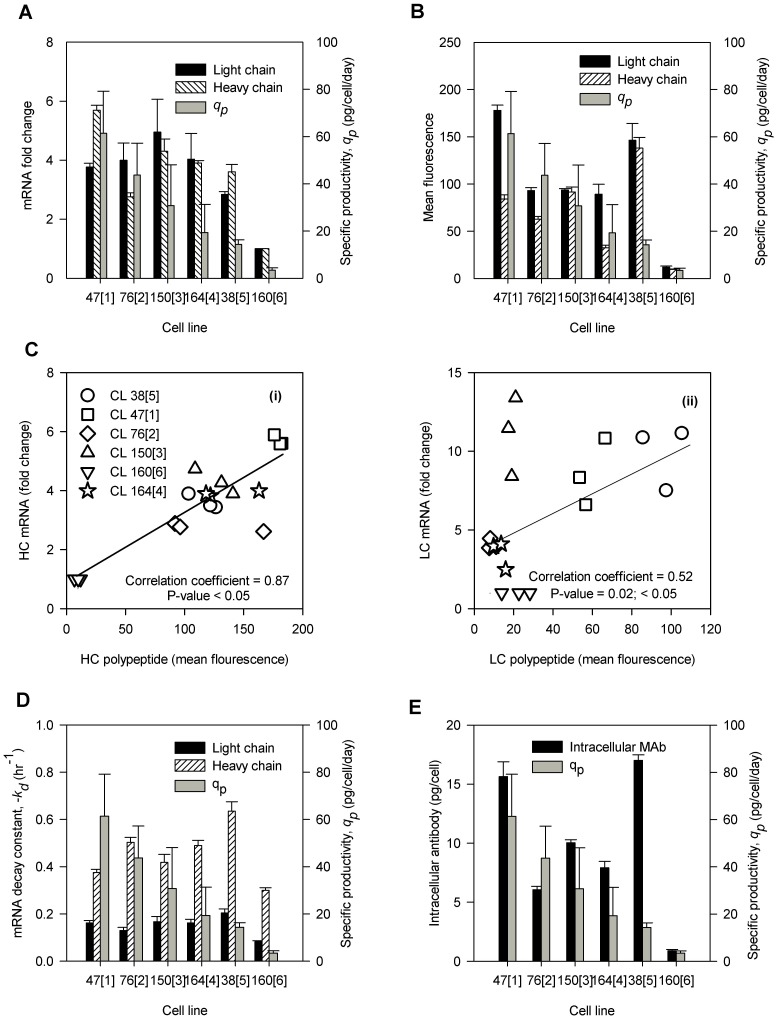
The relationship between mRNA and polypetides in 6 CHO cell lines. HC and LC mRNA levels (A; P-values<0.001), intracellular HC and LC polypeptides levels (B; P-values<0.001), mRNA degradation constant, -*k_D_* (D; P-values≤0.001) and intracellular MAb (E; P-value<0.001) at growth phase (day 3) of batch cultures. The correlation of mRNA level and polypeptides (C); (i) HC and (ii) LC. Error bars symbolize the standard deviation calculated from three biological replicates. P-values were calculated based on Student t-test analysis at 95% confidence intervals.

To determine if differences in specific productivity between the six GS-CHO cell lines are correlated to the relative content of mRNA, attempts were made to compare HC and LC mRNA levels by qPCR. Figure 4A demonstrates the levels of HC and LC mRNA. We have observed a good relationship between HC mRNA levels and specific productivity if we exclude CL 76[2] cell line. Likewise, LC mRNA displays a similar pattern of relationship in 150[3], CL 164[4], CL 38[5], and CL 160[6] but such relationship would disappear if we include the high producers, CL 47[1] and CL 76[2]. Pearson correlation coefficient analysis showed a positive correlation between specific productivity and the level of HC and LC mRNA in the cell lines with Pearson correlation coefficients of ρ = 0.70 and ρ = 0.62 (P-value<0.05), respectively. The LC to HC ratio of mRNA in the six cell lines are demonstrated in Table 2. The results indicate that, consistent to the ratio of LC to HC GCNs, LC to HC mRNA ratios show that LCs are also present abundantly in all six cell lines, though were found to be lower in CL 47[1] and CL 38[5].

Flow cytometric analysis was performed to determine whether the level of mRNA reflected the level of intracellular polypeptides. Figure 4B indicates the level of intracellular polypeptides in the six cell lines at growth phase. Generally, we observed a lower expression of HC polypeptide than of LC polypeptide. The HC polypeptide level in CL 47[1] did not reflect its high mRNA level. In CL 164[4], we observed a similar phenomenon but the difference was more severe. This could have resulted from a higher HC mRNA degradation rate in CL 164[4] compared to CL 47[1] (Figure 4D). In contrast, a lower mRNA HC level in CL 38[5] has resulted in higher polypeptide levels (relative to CL 47[1]). Therefore, the bottleneck that causes low productivity in CL 38[5] does not lie in protein translation.

Figure 4C shows the correlation between HC mRNA level and the HC polypeptides at growth phase (ρ = 0.87; P-value<0.05), which agrees with our previous observations in cytostatic system [16]. Unlike HC (Figure 4C(i)), LC mRNA and LC polypeptides exhibit a weaker correlation (ρ = 0.52; P-value<0.05) as indicated in Figure 4C(ii). Regardless of this correlation, the LC polypeptide was generally produced in excess, with LC to HC ratio of more than one as indicated in Table 2. One of the medium producers, CL 164[4] had a higher LC to HC polypeptide ratio. Besides, CL 150[3] and CL 38[5] showed the lowest LC to HC polypeptide ratio compared to the other cell lines. This could be one of the reasons for the lack of correlation between LC polypeptide and specific productivity (ρ = 0.11; P-value>0.05) compared to HC polypeptide (ρ = 0.67; P-value<0.05).

To determine the mRNA degradation rates in the six cell lines, Actinomycin D was added to cultures 30 hours after inoculation to inhibit transcription. The LC and HC mRNA degradation rates were plotted in Figure 4D. The HC mRNA decay rates were generally higher than LC mRNA in all cell lines. The HC mRNA decay rates of CL 76[2], CL 150[3] and CL 164[4] were 1.34, 1.11, and 1.31-fold higher than CL 47[1]. We observed stable HC and LC mRNA in CL 160[6] as indicated in Table 2 which corresponds to slower degradation rates (22 and 24% lower, respectively, than CL 47[1]). Interestingly, CL 38[5] showed the highest rates of degradation for HC and LC (1.69 and 2.88-fold higher than CL 47[1]). Besides, the correlation of mRNA decay rates with specific productivity appeared insignificant (LC: ρ = 0.21, P-value = 0.68; HC: ρ = −0.08, P-value = 0.88).

Figure 4E shows the level of intracellular antibody at growth phase as quantified by ELISA assay. The levels of intracellular antibody were related to the specific productivity in CL 47[1], CL 150[3], CL 164[4] and CL 160[6]. This is not consistent to CL 76[2] and CL 38[5]. Compared to CL 150[3], CL 76[2] exhibited 1.67-fold lower levels of intracellular antibody. The highest intracellular antibody levels, CL 38[5] were 1.08-fold higher than CL 47[1]. This could be one of the reasons that results in a weak correlation (ρ = 0.59, P-value<0.05) between intracellular antibody levels and specific productivity in the six cell lines as determined by the Pearson correlation coefficient analysis.

**Figure 5:**
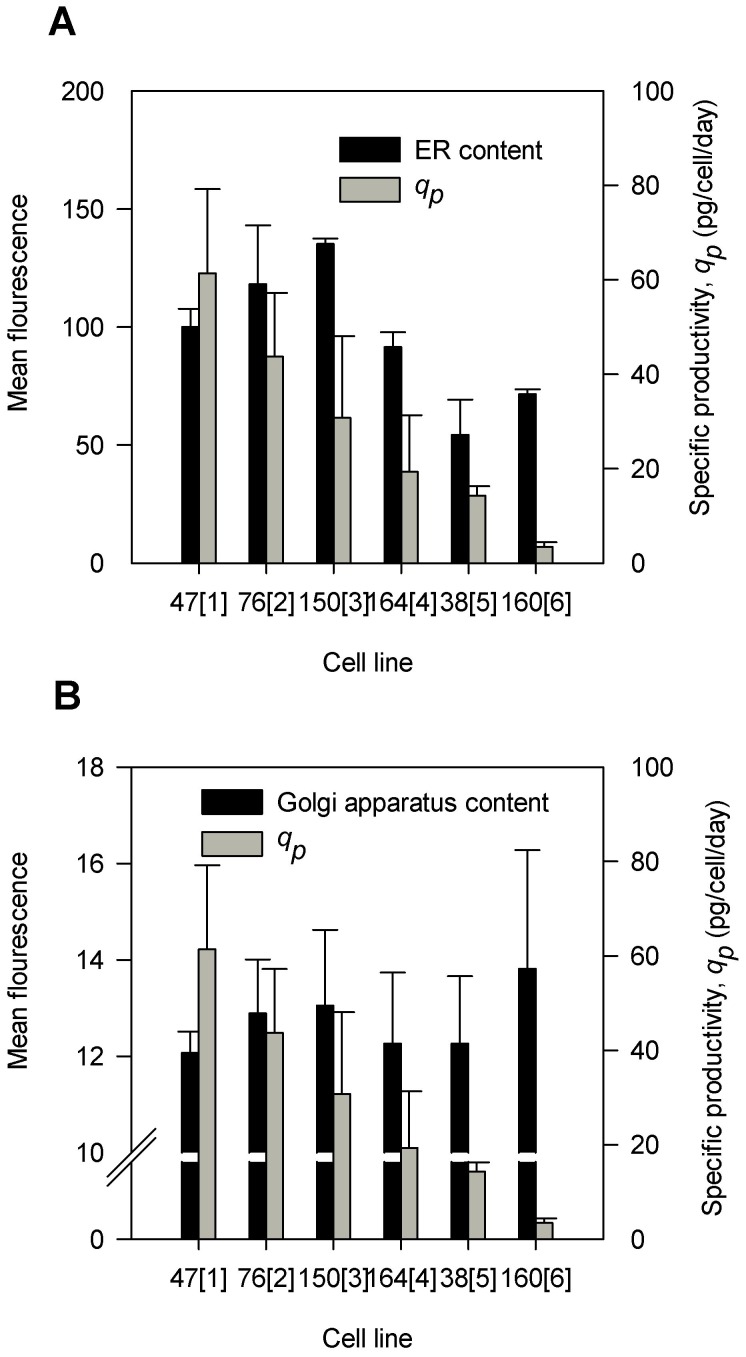
Flow cytometric analysis of endoplasmic reticulum (ER) and Golgi apparatus. ER (A; P-value<0.001) and Golgi apparatus (B; P-value<0.001) levels at growth phase (day 3) normalized based on the cell size as measured by flow cytometry analysis. Error bars depict standard error calculated from three biological replicates. P-values were calculated based on Student t-test analysis at 95% confidence intervals.

Flow cytometric analysis was performed to analyze the ER and Golgi apparatus contents in the six cell lines during growth phase. The ER contents, as indicated in Figure 5A were normalized based on cell size to exclude any size effects [51]. CL 150[3] showed the highest ER content compared to the other cell lines while CL 38[5] showed the lowest content. Pearson correlation coefficient analysis showed that there is no correlation between ER content and specific productivity (ρ = 0.06, P-value>0.05). Figure 5B showed the Golgi apparatus content in the six cell lines. These values were also normalized to the cell size. The Golgi apparatus content was found to be similar in most of the cell lines. However, CL 160[6] exhibited the highest level. Pearson correlation coefficient analysis showed that there is no correlation between Golgi apparatus content and specific productivity (ρ = −0.30, P-value>0.05).

### Correlation analysis of molecular markers during exponential phase

**Figure 6:**
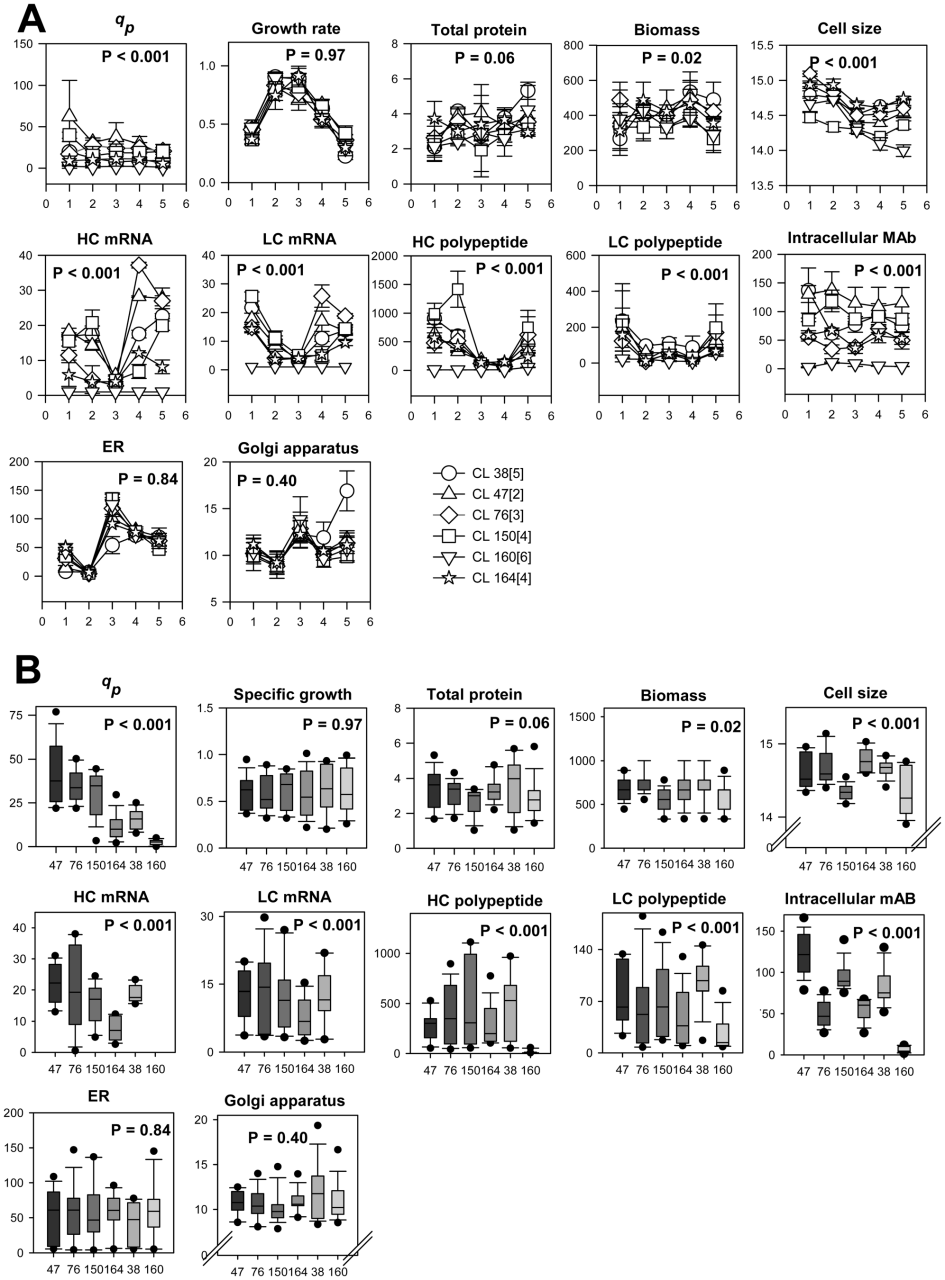
Data distribution of molecular markers of six GS-C cell lines. Data distribution collected throughout the growth phase (day 1 to day 5) of triplicate batch cultures in the six GS-CHO cell lines (A). P-values were calculated using one-pair ANOVA analysis. Boxplot edges represent quartiles of 25^th^ and 75^th^. The median, where 50 percent of data are distributed is represented by the line between the 25^th^ and 75^th^ quartiles Boxplot whiskers indicate maximum and minimum values in the distributions. Dashed line represents the whole population median (B).

To test the reliability or integrity of our data, we looked at measurement of molecular markers on a daily basis during the growth phase (days 1 to 5) of batch cultures as shown in Figure 6A. The distribution of data was visualised graphically using box plots as shown in Figure 6B which allows visualisation of the median, interquartile ranges, the outliers and the extreme values of the respective molecular markers. One-pair ANOVA analysis was employed to determine the significance of the differences of these molecular markers during the growth phase of batch cultures. We then used the expanded data to estimate the correlation coefficient, employing Pearson correlation coefficient analysis and the correlations generated from this analysis were tabulated in Table 3.

**Table 3:** Pearson correlation analysis of molecular markers.

Molecular marker(s)	Day 1	Day 2	Day 3	Day 4	Day 5
Growth rate	ρ = 0.27	ρ = −0.33	**ρ = −0.76**	ρ = 0.27	**ρ = 0.53**
	*P* = 0.29	*P* = 0.18	***P*** **<0.001**	*P* = 0.28	***P*** ** = 0.02**
Cell size	ρ = 0.10	ρ = −0.02	ρ = −0.08	ρ = 0.18	ρ = 0.28
	*P* = 0.70	*P* = 0.45	*P* = 0.75	*P* = 0.48	*P* = 0.27
Total protein	ρ = 0.22	ρ = −0.04	ρ = 0.12	ρ = −0.09	ρ = 0.13
	*P* = 0.39	*P* = 0.87	*P* = 0.63	*P* = 0.71	*P* = 0.60
Biomass	ρ = 0.17	**ρ = −0.49**	**ρ = 0.49**	ρ = 0.22	ρ = 0.10
	*P* = 0.50	***P*** ** = 0.04**	***P*** ** = 0.04**	*P* = 0.38	*P* = 0.71
Endoplasmic reticulum	**ρ = −0.54**	ρ = −0.22	ρ = 0.06	**ρ = 0.56**	ρ = −0.17
	***P*** ** = 0.02**	*P* = 0.38	*P* = 0.82	***P*** ** = 0.02**	*P* = 0.49
Golgi apparatus	ρ = −0.05	ρ = −0.02	ρ = −0.30	ρ = −0.03	ρ = −0.10
	*P* = 0.85	*P* = 0.92	*P* = 0.23	*P* = 0.92	*P* = 0.70
Heavy chain mRNA	**ρ = 0.82**	**ρ = 0.54**	**ρ = 0.70**	**ρ = 0.73**	**ρ = 0.78**
	***P*** **<0.001**	***P*** ** = 0.02**	***P*** **<0.001**	***P*** **<0.001**	***P*** **<0.001**
Light chain mRNA	**ρ = 0.61**	**ρ = 0.57**	**ρ = 0.62**	**ρ = 0.73**	**ρ = 0.82**
	***P*** ** = 0.01**	***P*** ** = 0.01**	***P*** ** = 0.01**	***P*** **<0.001**	***P*** **<0.001**
Heavy chain polypeptide	**ρ = 0.53**	ρ = 0.40	**ρ = 0.67**	ρ = 0.14	**ρ = 0.73**
	***P*** ** = 0.02**	*P* = 0.10	***P*** **<0.001**	*P* = 0.57	***P*** **<0.001**
Light chain polypeptide	ρ = 0.31	ρ = 0.07	ρ = 0.11	ρ = −0.02	**ρ = 0.67**
	*P* = 0.21	*P* = 0.79	*P* = 0.66	*P* = 0.95	***P*** **<0.001**
Intracellular Mab	**ρ = 0.61**	**ρ = 0.72**	**ρ = 0.59**	**ρ = 0.63**	**ρ = 0.63**
	***P*** ** = 0.01**	***P*** **<0.001**	***P*** ** = 0.01**	***P*** **<0.001**	***P*** **<0.001**

Analysis of Pearson correlation coefficient (ρ) of molecular markers during growth phase (day 1 to day 5). Highlighted data are molecular markers with P-value≤0.05.

The one-pair ANOVA analysis showed that the difference in the specific productivity in the six cell lines throughout the growth phase is significant (P-value<0.001). The medians of CL 47[1], CL 76[2] and CL 150[3] in the specific productivity plot are 46.5, 32.4 and 34.7 pg/cell/day respectively which fell above the median line of specific productivity plot. However, this was not observed in CL 164[4], CL 38[5] and CL 160[6] where the medians are 13.7, 15.8 and 2.5 pg/cell/day, respectively which fell below the median line of productivity plot. We also observed a broader distribution of specific productivity in CL 47[1], CL 76[2] and CL 150[3] compared to CL 164[4], CL 38[5] and CL 160[6] which suggests that the specific productivity in three highest producers varied at a higher degree than that of the three lowest producers during the growth phase of batch cultures.

As for the physiological characteristics, only biomass and cell size were found to be significantly different (P-value<0.05) while the difference in the specific growth rates and total protein content were observed insignificant (P-value>0.05). The cell size distributions in the six cell lines are broader than the biomass distributions. As indicated in Table 3, we observed the absence of correlation between these molecular markers and specific productivity in most of the days from day 1 to day 5 of batch cultures. The correlation between biomass and specific productivity only appeared on day 2 and 3. Meanwhile, the correlation between growth rate and specific productivity were only visible on days 3 and 5.

At the molecular level, the levels of mRNA, polypeptides and intracellular antibody were found to be significantly different (P-value<0.05). This was not observed in ER and Golgi apparatus content (P-value>0.05). A greater variability was observed in the mRNA and polypeptide expression of HCs and LCs from the large interquartile ranges (25^th^ to 75^th^ quartiles), which suggests that the expression of mRNA and polypeptides varied greatly throughout the growth phase. The interquartile ranges were also largely overlapped within the cell lines. Interestingly, the large overlap of interquartile ranges were not seen in the intracellular antibody distributions indicating that the variability in the level of intracellular antibody throughout the growth phase occurred at a lower degree. Between these molecular markers, only HC mRNA, LC mRNA and intracellular antibody were found to correlate to the specific productivity and these correlations appeared consistently from day 1 to day 5 of growth phase as showed in Table 3. Meanwhile, the correlations between polypeptides and specific productivity appeared only on certain days; HC at day 1, day 3 and day 5 while LC at day 5.

## Discussion

There are seemingly contradictory findings in the literature as to the relationship between growth rate and productivity in mammalian cell culture. Growth rate has not only been shown to be positively or negatively correlated to productivity by different workers but has also been shown to have no correlation by others [52]. Growth rate related productivity could vary with cell line, the nature of the recombinant gene expressed or the promoter/enhancer used to generate product expression [52]. Nevertheless, some results do directly conflict. In our study, changes in specific productivity were apparently negatively associated with the specific growth rate. The negative association presumably resulted as a consequence of the utilisation by highly proliferating cells of a large proportion of available substrates for biomass synthesis and only a small proportion will be directed to recombinant product synthesis.

In this study, neither total protein content nor cell size was found to be correlated to specific productivity. In a study using centrifugal elutriation to sort cells according to their size and cell cycle phase [53], the higher productivity was due to the increase in cell size as the cells progressed from S phase to G_2_/M phase. It may be that differences in size between clones are not significant enough to explain the differences in productivity and that size is governed by the cell cycle phases which in turn are not proportionally different in different cell lines. The results also suggests that total protein content does not necessarily have to correlate to the cell size [16] for enhanced specific productivity.

The normalization of specific productivity based on physiological characteristics has proven beneficial to our study by unveiling the limitation in CL 47[1]. As the cells progressed to the stationary phase (day 5) of batch culture, the production of monoclonal antibody per unit mass in CL 47[1] did not change proportionally compared to the other cell lines. This suggests that saturation in the protein synthesis pathway may have occurred in this cell line as a result of increased specific productivity. The post-transcriptional mechanisms such as translation, protein folding and assembly become the limiting factor in high producing cells that continue to transcribe HC and LC mRNA at a high level. Unlike CL 47[1], CL 150[3] which has smaller size and lower cell mass (total protein and biomass) showed a proportional increase of specific productivity throughout the growth phase.

Our observation that a high HC and LC gene copy number does not result in high productivity was consistent with previous reports [11], [54] It appears that this lack of correlation between gene copy number and productivity could arise from the chromosomal rearrangements caused by the high rates of homologous recombination and translocation [9]. During chromosomal rearrangements, the affected gene could be placed near or in heterochromatin thus inducing inappropriate gene inactivation [55]. Nevertheless, the specific productivity was found to be correlated to mRNA levels. A decrease in HC and LC mRNA levels resulted in a proportional decrease in the specific productivity with the notable exception of HC mRNA in CL 76[2] and LC mRNA in CL 47[1] and CL 76[2]. A strong relationship between mRNA levels and recombinant protein production was found in the low-producing cell lines [56]. This relationship was visible until a threshold was reached from which an increase in mRNA level did not result in a further increase in the specific productivity. Beyond this threshold, the translational/secretory machinery of the cells becomes the major determinant of antibody production [56]. Moreover, the findings of the current study are consistent with previous study [17] which found a greater correlation between HC mRNA and specific productivity than that obtained with LC mRNA, and a lack of correlations between the ratio of LC to HC mRNA and specific productivity. The better correlation obtained between the level of HC mRNA and HC polypeptide than between LC mRNA and LC polypeptide suggests that the translation of LC could limit specific productivity. Regardless of these correlations, the LC polypeptide was generally produced in excess to that of HC polypeptide with a LC to HC ratio of higher than one. This could be due to the faster translation rate of LC than HC [57]. Even though an excess of LC polypeptide is necessary for efficient assembly of antibody, this is not sufficient for enhanced specific productivity [58]–[60]. This has become obvious in CL 164[4], in which a LC to HC ratio of 2.78 did not result in high specific productivity.

The high level of intracellular antibody in CL 38[5] was resulted from the increased GCN, mRNA and polypeptide levels. However, these increased levels did not reflect the specific productivity suggesting that the low specific productivity in CL 38[5] could have resulted from a slow antibody secretion rate [17]. The flow cytometric analyzes of ER and Golgi apparatus content provide a better understanding of the limiting factors that can cause a decrease in productivity in CL 38[5]. Interestingly, with similar Golgi apparatus capacity, ER content was lower in CL 38[5] (50% lower than 47[1]). This reduced capacity appeared to accommodate a large amount of HC and LC polypeptides for folding and assembly. However, the high intracellular antibody which could result from an efficient processing machinery in the ER, was only secreted partially, suggesting the possibility of antibody aggregation in the ER [34]. This was supported by a much higher HC and LC mRNA degradation rate may which have occurred in CL 38[5] and could indicate a reduced translation activity to avoid congestion in the ER. It may probably that even if the assembly is efficient in CL 38[5], the transport from ER to Golgi to secretion may become the limiting factor thus reducing secretion [61], although this interpretation cannot entirely be deduced from the data shown.

Using Pearson correlation coefficient analysis helped us to identify the molecular markers that can best represent the relation with productivity. We have shown that, at the mid-exponential phase, several molecular markers (growth rate, ρ = −0.76; biomass, ρ = 0.49; HC mRNA, ρ = 0.70; LC mRNA, ρ = 0.62; HC polypeptide, ρ = 0.67; intracellular MAb, ρ = 0.59) appeared to correlate well with specific productivity. However, the reliability of these molecular markers to predict productivity remains in question. Some of these correlations were reported before, while some were not. The striking feature of the previous studies is their inconsistency which suggests that some of the correlations could be rather coincidental or may only appear when substantial differences in the culture conditions or treatments are generated (such as culturing cells in low temperature or high osmolarity). To improve the reliability of molecular markers, daily measurements during the growth phase was shown to improve the reliability in productivity studies. Above all the molecular markers cell size, total protein and Golgi apparatus content, did not show any correlation to the specific productivity as indicated by the absence of correlation throughout the culture duration. Specific growth rate, biomass, ER and LC polypeptide were only correlated to specific productivity at certain time points during the growth phase. The positive correlation between HC mRNA, LC mRNA, HC polypeptides and intracellular antibody with specific productivity appeared consistently throughout the growth phase of batch culture which are generally in agreement with previous reports on the cellular mechanisms that dictate relationships between productivity and mRNA levels in various cell lines [9], [12]–[14], [16], [17], [20], [62]. This quantitative correlation suggests that HC and LC mRNA chains is extraordinary stable and that they appear to be translated with equal efficiency during the culture duration. This result also indicates that HC and LC mRNA processing and to some extent RNA stability are the most important regulatory processes determining monoclonal antibody level.

## Conclusion

An enhanced productivity can result from various alterations that occur at transcriptional, translational and post-translational levels during the synthesis of monoclonal antibody. It is important to understand such mechanisms to allow future improvements in cell productivity. In this study, we provide a better understanding of the bottlenecks in antibody production that can cause decreased productivity in GS-CHO cell lines.

The reduced productivity resulted from decreased mRNA levels and slow secretion rates of antibody. In one of the high-producing cells, 47[1], productivity is limited by the saturation of antibody production during protein synthesis. The determination of HC and LC mRNA, HC polypeptides and intracellular antibody can be considered an accurate measure of antibody secretion. This method of quantitation is suitable for obtaining reliable estimates of productivity in CHO clones. Moreover, the measurement of molecular markers on a daily basis during the growth phase of batch cultures ensures reliable and consistent analysis identifying differences in productivity levels in mammalian cell cultures.
